# A Review of the Multidisciplinary Diagnosis of Interstitial Lung Diseases: A Retrospective Analysis in a Single UK Specialist Centre

**DOI:** 10.3390/jcm5080066

**Published:** 2016-07-27

**Authors:** Nazia Chaudhuri, Lisa Spencer, Melanie Greaves, Paul Bishop, Anshuman Chaturvedi, Colm Leonard

**Affiliations:** 1 North West Interstitial Lung Disease Unit, University Hospital of South Manchester, Wythenshawe Hospital, Southmoor Road, Wythenshawe, Manchester M23 9LT, UK; Melanie.Greaves@uhsm.nhs.uk (M.G.); paul.bishop@uhsm.nhs.uk (P.B.); antshuman.chaturvedi@uhsm.nhs.uk (A.C.); Colm.Leonard@uhsm.nhs.uk (C.L.); 2 Aintree Chest Centre, University Hospital Aintree, Lower Lane, Liverpool L9 7AL, UK; Lisa.Spencer@aintree.nhs.uk

**Keywords:** multidisciplinary team, diagnosis, interstitial lung disease, idiopathic pulmonary fibrosis

## Abstract

The accurate diagnosis and management of individuals with interstitial lung diseases (ILDs) poses an interesting challenge in clinical practice. A multidisciplinary team (MDT) approach is considered the gold standard. This is a single-centre retrospective review spanning a five-year period. We assessed the accuracy of prior ILD diagnosis, the methodology used to establish a correct diagnosis and how an MDT approach affected subsequent management. Our data supports an MDT approach in an experienced specialist ILD centre. We have demonstrated that diagnosis is often changed after an MDT review and that this impacts the subsequent management. Our results demonstrate that an MDT approach to diagnosis can establish a diagnosis in the majority of cases when prior diagnosis is uncertain (76%). We also show that a prior diagnosis of idiopathic pulmonary fibrosis is deemed inaccurate in over 50% of cases after MDT discussion. We have shown that during diagnostic uncertainty the considered gold standard of proceeding to a lung biopsy is not always feasible due to disease severity and comorbidities. In these circumstances, an MDT approach to diagnosis of ILDs combines clinical data with serial lung function and disease behavior, with or without responses to previous treatment trials to establish an accurate expert diagnosis.

## 1. Introduction

Interstitial lung diseases (ILD) are a group of over 100 heterogeneous diseases [1]. The most common idiopathic ILD is idiopathic pulmonary fibrosis (IPF) and it has a median life expectancy of three to five years from diagnosis [2] with a prevalence that is increasing by 5% per year [3,4]. Accurate and early diagnosis of an ILD is paramount for patient and clinician as it predicts prognosis and allows accurate targeting of the available treatment modalities, whether immunosuppression, anti-fibrotic therapies, lung transplantation or involvement in clinical trials.

Consensus discussions between multidisciplinary team (MDT) members have been shown to increase diagnostic accuracy and confidence of idiopathic interstitial pneumonias (IIP) [5]. In this study [5], expert respiratory clinicians, thoracic radiologists and pathologists were provided with clinical information in a step-wise sequential manner for a number of IIPs and they were asked to give their diagnosis and the confidence level of their diagnosis. The more information they were provided with, combined with the dynamic interactions and discussions between the MDT members, increased interobserver agreement and diagnostic accuracy, with histopathological information having the greatest impact on final consensus diagnosis. In a further study [6], the diagnostic agreement and accuracy between different MDTs has been demonstrated to be good and is highest for a diagnosis of IPF but lower for hypersensitivity pneumonitis (HSP) compared to individual clinicians or radiologists. An MDT approach to diagnosis and management of ILD is therefore now considered the gold standard and an integral part of ILD management and guidelines [7,8,9,10]. ILD MDTs consisting of expert respirologists, radiologists and histopathologists have been shown to minimise interobserver variation and improve diagnostic confidence [5,6].

We are a large tertiary referral centre for ILD cases in the northwest of England and we support a population of five million people. We have been performing a multidisciplinary ILD meeting to discuss selected newly referred clinical cases from the region for almost a decade. Our clinical database has over 900 patients and in 2014 we saw over 350 new referrals. The development of specialist commissioning for ILD in England, the establishment of dedicated ILD centres and the approval of anti-fibrotic therapies has resulted in this number of new referrals increasing by 25% year on year. The aims of this study were to reinforce the importance of an MDT approach to diagnosis. Our objectives were to explore the accuracy of prior ILD diagnosis from referral centres that did not utilise MDT approaches for diagnosis. After a single-centre MDT discussion of clinical cases, we aimed to explore how these diagnoses are achieved and the influence of MDT discussion on subsequent management. Here we present a retrospective review of our MDT reviews.

## 2. Methods

This is a single-centre retrospective review of electronic patient letters and MDT records for a five-and-a-half-year time period spanning February 2005 to June 2008 (three years, four months) and January 2011 to February 2013 (two years, one month). Data from July 2008 through to December 2012 was not available as MDT data and patient letters were not available electronically for review. Our MDT consists of two consultant respiratory physicians with a specialist interest and expertise in ILD, performing initially monthly but since January 2013 weekly dedicated ILD MDTs in our centre, a thoracic radiologist with expertise in ILD, a thoracic histopathologist, an ILD specialist nurse, an MDT coordinator and, more recently, an ILD pharmacist. We receive tertiary referrals from respiratory physicians in local hospitals within the northwest of England, spanning a population of five million people. Patients with a prior diagnosis of ILD based on clinical history and high resolution computerised tomography (HRCT) imaging plus or minus surgical lung biopsy are referred for expert multidisciplinary review for a variety of reasons including uncertainty of diagnosis, failure to respond to standard therapy, assessment for initiation of second-line immunosuppression or anti-fibrotic therapy, consideration for clinical trials and lung transplantation. Between February 2005 and December 2012 we conducted eight MDT meetings per year and due to clinical demand this increased to a weekly meeting from January 2013. MDT discussions between members of the team were conducted with all available clinical information including serological data, HRCT images and bronchoalveolar lavage and surgical lung biopsy data when available. The members of the MDT were not blinded to any data and the MDT discussions were performed collectively with all data as would occur in real world clinical settings. All MDT data was collected and recorded on a locally devised ILD MDT proforma that detailed patient demographics, clinical history, diagnosis prior to referral, MDT discussion of imaging and histopathology (if available) and final MDT diagnosis and management plan. This allowed us to analyse a number of key questions:

1. How often does an MDT review of clinical cases alter the diagnosis of ILD?
2. How often is this change in diagnosis based on radiographic imaging alone or combined computerised tomography (CT) imaging and biopsy?
3. How does this change in diagnosis subsequently alter patient management?
4. Has there been a temporal change in the management of cases in the two time periods?

## 3. Results

**Figure 1:**
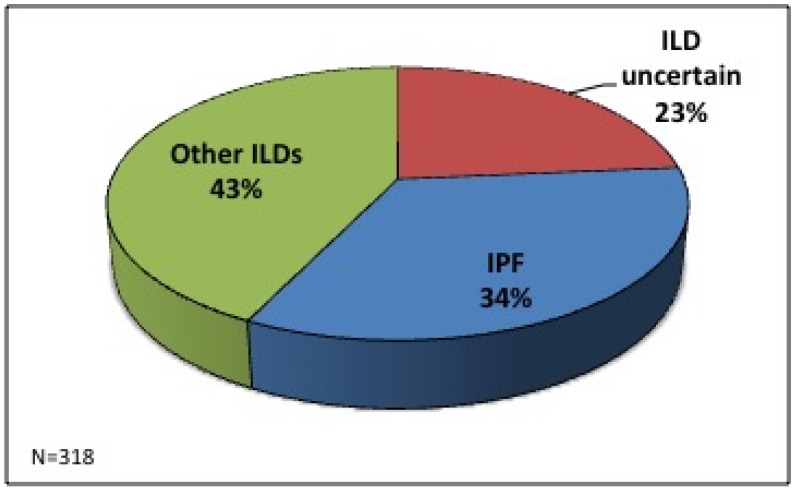
The initial reasons for referral to the interstitial lung disease multidisciplinary meeting.

A total of 318 clinical cases were discussed in our ILD MDT in this time period (*n* = 165 between February 2005–June 2008 and *n* = 153 between January 2011–February 2013). Seventy-five (24%) cases were referred because of an ILD of unknown classification, 107 (33.5%) were referred with a prior diagnosis of IPF and 136 (42.5%) were referred with other ILD diagnoses (non-specific interstitial pneumonitis (NSIP), connective tissue disease–related ILD (CTD-ILD), sarcoidosis, hypersensitivity pneumonitis (HP) and other ILDs) (Figure 1). Between 2005 and 2008 the majority of the MDT discussions were based on radiographic imaging alone (91%). This is compared to 62% between 2011 and 2013. There was a 31% increase in MDT discussions involving combined radiology imaging and histopathological biopsy between the two time periods (2005 to 2008 vs. 2011 to 2013).

### 3.1. MDT Discussion of ILD of Unknown Classification

Seventy-five cases were referred to our MDT because the referring physician was unable to classify the type of ILD. Our MDT discussion was able to make a consensus ILD diagnosis in 57 of 75 (76%) of cases. In 2005 to 2008 the majority of these consensus diagnoses were based on radiological imaging alone (42 of 44, 95%) compared to 16 of 31 (52%) between 2011 and 2013. There was a 43% increase in MDT discussions of unclassifiable ILDs involving both radiology and histopathological biopsy between the two time periods (2005 to 2008 vs. 2011 to 2013).

**Figure 2:**
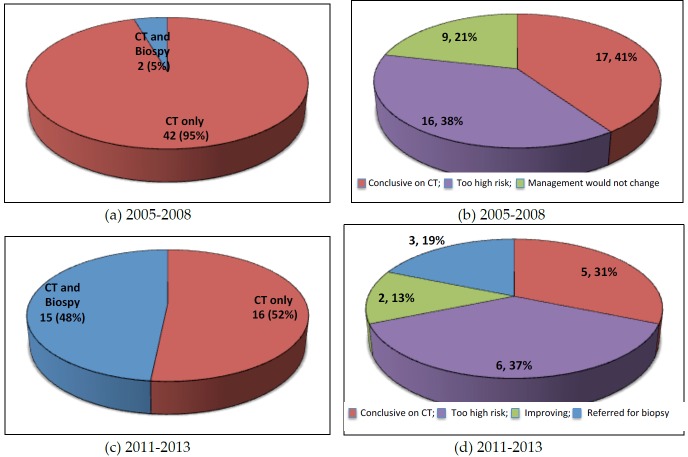
The multidisciplinary discussion and diagnosis of interstitial lung disease of unknown classification: (**a**) The modalities used to make the diagnosis in 2005–2008; (**b**) The reasons why biopsy was not performed when diagnosis was made by CT imaging; (**c**) The modalities used to make the diagnosis in 2011–2013; (**d**) The reasons why biopsy was not performed if diagnosis was made by CT imaging alone.

Between 2005 and 2008, 42 of 44 (95%) of the diagnoses were based on radiological imaging alone (Figure 2a). Of these, 17 (41%) were deemed to be conclusive by CT imaging. Of the remaining 25 patients, biopsy was not performed because it was deemed too high risk in 16 (38%) patients (average diffusing capacity of the lung for carbon monoxide (DLCO) of 35%) and in nine (21%) patients it was deemed that clinical management would not be altered after a biopsy (Figure 2b). Between 2011 and 2013, 16 of 31 (52%) of the diagnoses were based on radiological imaging alone (Figure 2c). Five (31%) were deemed conclusive on CT imaging alone, six (37%) patients were deemed too high risk to proceed to biopsy, two (13%) patients were asymptomatic or improving and three (19%) patients were referred for surgical biopsy to clarify a diagnosis (Figure 2d).

MDT discussion resulted in a change of treatment in 30 (40%) cases. This included starting or discontinuing immunosuppresant therapies. Thirty-one (41%) cases had no treatment change and data regarding treatment alterations was not available in 14 cases.

### 3.2. MDT Discussion of IPF

**Figure 3:**
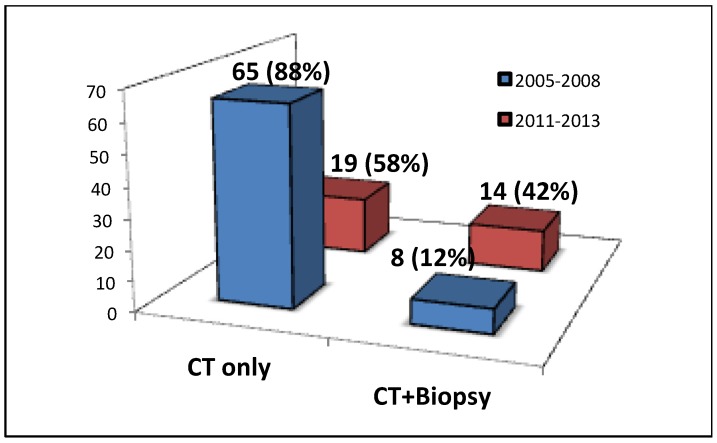
The modalities utilised to make a multidisciplinary diagnosis of idiopathic pulmonary fibrosis in 2005–2008 and 2011–2013.

One hundred and seven cases were referred to our specialist ILD service with a prior diagnosis of IPF based on clinical and HRCT parameters. After MDT discussion this diagnosis was deemed correct as defined by a definite usual interstitial pneumonia (UIP) pattern [6] in 50 (47%) cases and incorrect in 57 (53%) cases. Between 2005 and 2008, 65 of 73 (88%) IPF diagnoses were based on CT imaging alone. Between 2011 and 2013 this reduced to 19 of 33 (58%). There was a 30% increase in MDT discussions involving both radiology and histopathological biopsy between these two time periods (Figure 3).

**Figure 4:**
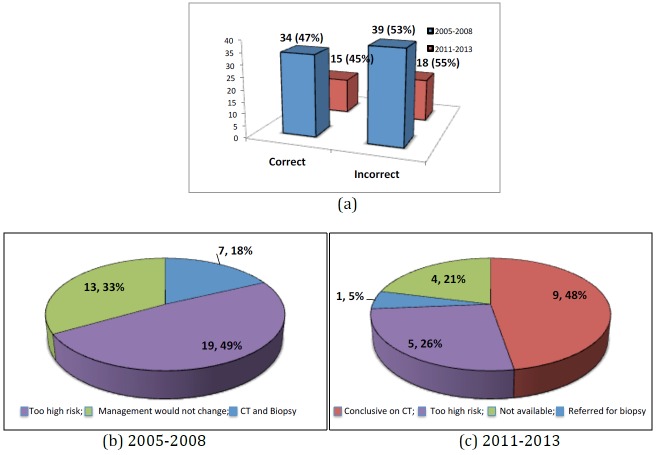
The multidisciplinary diagnosis of idiopathic pulmonary fibrosis. (**a**) The accuracy of diagnosis of idiopathic pulmonary fibrosis in 2005–2008 and 2011–2013; (**b**) The diagnosis of fibrotic NSIP in 2005–2008 and why biopsy was not performed; (**c**) The diagnosis of fibrotic NSIP in 2011–2013 and why biopsy was not performed.

The most common alternative diagnosis was that of fibrotic NSIP. In 2005 to 2008, 39 patients were deemed to have an incorrect diagnosis of IPF (Figure 4a). Seven (18%) were confirmed to have fibrotic NSIP by CT imaging and biopsy. In 32 (82%) cases a diagnosis of fibrotic NSIP was based on clinical course and radiological imaging alone. This was because 19 (49%) patients were deemed too high risk for biopsy with an average DLCO of 38.7%. In 13 (33%) patients it was felt that biopsy would not change clinical management (Figure 4b). In 2011 to 2013, 18 patients were deemed to have an incorrect diagnosis of IPF (Figure 4). Five (28%) had alternative ILD diagnoses and 13 (72%) were diagnosed as fibrotic NSIP based on CT imaging and a more stable clinical course. Of all cases in which the diagnosis was based on CT imaging alone (19 of 53), nine (47%) were deemed correct by CT, five (26%) were deemed too high risk to biopsy, one (5%) patient was referred for a surgical biopsy and data was not available in four (21%) (Figure 4c).

MDT discussion resulted in a change of treatment in 53 (50%) cases. The main treatment changes were stopping immunosuppressant therapies and commencing pirfenidone. Thirty-six (34%) cases had no treatment change and data was not available in 18 cases.

### 3.3 MDT Discussion of Other ILDs

One hundred and thirty-six patients were referred with other ILD diagnoses. After MDT discussion, the diagnosis was correct in 91 of 136 (67%) of the cases and incorrect in 45 of 136 (33%). Between 2005 and 2008, 44 of 47 (94%) of the diagnoses were based on CT imaging alone. Between 2011 and 2013 this was reduced to 64%. There was a 30% increase in discussions involving both radiology and histopathological biopsy between these two time periods.

Between 2011 and 2013, consensus diagnosis was achieved in 27 (47%) patients by CT imaging alone, six (11%) patients were referred for surgical biopsy and seven (12%) were deemed too high risk for biopsy. In 13 (23%) patients, biopsy was deemed not to change management and one (2%) patient declined biopsy. Data was not available for three cases.

MDT discussion resulted in a change of treatment, specifically starting or discontinuing immunosuppressant therapies, in 53 (39%) cases. Sixty-three (46%) cases had no treatment change and data regarding treatment change was not available in 20 cases.

## 4. Discussion

This is a single-centre retrospective review of MDT data from a large teaching university hospital based in the northwest of England. As far as we are aware, this is the largest published retrospective review of ILD MDT data. We have been conducting MDT meetings discussing referred ILD cases from the northwest region and have almost a decade of expertise in managing patients with ILD. Here we present a review spanning five-and-a-half years of available data collection.

Cases are referred to our centre from local hospitals that rely solely on general radiologists (i.e., not specifically thoracic-trained) reporting HRCTs, without MDT discussions, to make an ILD diagnosis. The interobserver agreement between individual radiologists in diagnosing ILDs, specifically IPF, has been demonstrated to be fair to moderate [11] and physicians in the community are more likely to disagree with ILD diagnosis than those in expert academic centres [12]. Current guidelines for diagnosis and management of ILDs therefore advocate a multidisciplinary team review as the gold standard [7,8,9,10]. The aims are to raise the standard of care for patients with ILDs and optimise diagnosis and management of this group of conditions. Our data supports these recommendations by highlighting an inaccuracy of prior diagnoses based on our revision of ILD diagnoses after a comprehensive MDT review.

Even within a specialist ILD centre, the financial constraints within the National Health Service in the United Kingdom denoted that MDT meetings were performed on a good will basis to address a clinical need and demand. As a result, in our early clinical practice only a select proportion of patients referred to our ILD centre were discussed in an MDT meeting. The ILD physicians selected cases according to the complexity and uncertainness of ILD diagnosis. Cases that were deemed by the ILD physician as conclusive on HRCT were not discussed. This is a major limitation of this retrospective review in that the case mix discussed in an MDT is not complete and is biased by selection. Despite this limitation, our clinical patient database demonstrates that the patients that were discussed in our MDT meetings are representative of the total new referrals that we received. Since the designation of the specialist ILD status in 2013, we now perform weekly MDT meetings discussing every patient referred to our center.

Approximately one-quarter of cases are referred as ILD of unknown classification. After MDT discussion of cases, we can reach a consensus and unified diagnosis in three-quarters of cases. Approximately 8% of total cases referred to our MDT remain unclassifiable which is comparable to published data [13]. Over time there has been a paradigm shift and increased utility of combined radiology and histopathological biopsies to achieve this consensus diagnosis. We feel this increase is a reflection of the biopsy recommendations in the ILD guidelines. These guidelines advocate the need for tissue biopsy when there is diagnostic uncertainty [7,8]. Despite this increase in the number of biopsies performed over time, the majority of patients are deemed too high risk, due to the severity of their disease as pertained by their poor lung function, or the presence of co-existing comorbidities. This is reflective on the fact that patients with ILDs tend to present in their later decades.

Guidelines specifically for the diagnosis and management of IPF advocate performing surgical lung biopsy in cases of possible usual interstitial pneumonitis (UIP) or those with atypical features on HRCT, followed by an MDT discussion to confirm the diagnosis [7]. However, lung biopsy is not without risk [14] and our data [15] and experts within the IPF field acknowledge that, often, a lung biopsy is not possible due to patient factors such as severity of fibrosis, comorbidities and patient choice [16,17,18]. In our real world setting we have observed a paradigm shift from over-reliance of HRCT and, specifically, lung biopsy to make an MDT diagnosis of IPF with increasing emphasis put on a working diagnosis of IPF based on clinical disease behavior over time and responses to therapy in those with a possible UIP pattern. These factors influence why the majority of the MDT discussions in this study principally involve clinical information, serological results, HRCT and, when available, bronchoalveolar lavage with less reliance on surgical lung biopsy results. 

A third of cases discussed in our ILD MDT had a prior clinical diagnosis of IPF. As discussed previously and in line with international guidelines [7], there has been a shift in time of increased utility of combined CT imaging and histopathological biopsy to achieve a diagnosis of IPF. Despite this increase over time, as previously discussed, the majority of patients were deemed too high risk to proceed with a surgical lung biopsy. Of particular concern, in our study, over half the IPF diagnoses are changed after ILD MDT review due to the absence of a definite UIP pattern on HRCT in those who did not have a surgical lung biopsy. Local radiologists in our region would overcall the presence of honeycombing on HRCT images 50% of the time. This is corroborated by a study that demonstrated that interobserver agreement of the CT criteria for UIP is only moderate, even in experienced thoracic radiologists [19]. The most common alternative differential diagnosis after MDT discussion was fibrotic NSIP. The difficulty, however, was that without the availability of a surgical lung biopsy in the majority of these cases, it was difficult for the MDT to distinguish between IPF and fibrotic NSIP in almost 50% of cases. In this situation, the MDT would rely more on additional clinical information such as evidence of stability on serial lung function monitoring before or after immunosuppressive therapy to consider a diagnosis of fibrotic NSIP to be most likely. The major limitation of this MDT strategy is that a diagnosis of fibrotic NSIP should only be conclusively made on biopsy, and stability in lung function can also be a feature of IPF due to the heterogeneity of its clinical course. Subsequent data from our group on a select cohort of patients diagnosed as fibrotic NSIP has demonstrated that the age of the patient, the decline in lung function over time and the failure to respond to immunosuppressive therapies within an MDT discussion are important factors used to make a working diagnosis of IPF when biopsy is not feasible [15].

This data highlights the difficulties often posed by the combination of the presence of comorbidities in an older population and the problems posed by delayed diagnosis. Symptoms can be present for many years before diagnosis and, thus, patients often present with more severe disease. These factors impact the suitability of patients for lung biopsy when diagnostic uncertainty ensues and go some way to explain the over-reliance on CT imaging and low biopsy referrals in these results. This data also represents the era prior to anti-fibrotic approval, when immunosuppression was the only available treatment versus supportive care for both IPF and fibrotic NSIP. Biopsy was principally required to distinguish between IPF and fibrotic NSIP and so, often, a clinical decision was made that biopsy would not alter management and therefore was not performed.

Change of diagnosis after MDT discussion is a recurring theme when addressing other ILDs. The diagnostic accuracy is somewhat better prior to MDT discussion compared to IPF. A third of diagnoses are changed after MDT discussion compared to over half in IPF.

Overall, for all cases, MDT discussion with subsequent diagnosis clarification resulted in a change in treatment which consisted of stopping or starting immunosuppressants or the introduction of the ant-fibrotic pirfenidone for IPF.

## 5. Conclusions

The accurate diagnosis and management of individuals with ILD poses an interesting challenge in clinical practice. ILD guidelines advocate an MDT approach to improve diagnostic accuracy and access to specialised treatments, with the ultimate goal of ensuring equality and improving patient care. ILD MDT diagnosis has also demonstrated better survival compared to patients diagnosed without MDT discussion [20]. Our data supports an MDT approach in an experienced specialised ILD center. We have demonstrated that diagnosis is often changed after an MDT review and that this impacts subsequent management. We have shown that, during diagnostic uncertainty, the considered gold standard of proceeding to a lung biopsy is not always feasible due to disease severity and comorbidities. In these circumstances, an MDT approach to diagnosis of ILDs combines clinical data with serial lung function and disease behaviour, with or without responses to previous treatment trials, to establish an accurate expert diagnosis. We acknowledge the major limitations of this retrospective review. We are presuming that a decade of experience in the diagnosis and management of ILDs serves to provide our MDT meetings with an expertise that is both robust and accurate. In the real world setting, we have developed collaborative bench-marking peer review strategies to address this on a local scale by developing a Northern ILD network. This review is of real-life clinical care and thus lacks the corroborative independent review of our clinical cases by a second independent blinded MDT. Despite this limitation, we feel we have demonstrated the importance of an MDT review to ensure accurate diagnosis, assessment and subsequent treatment, and we advocate and support the recommendation that a multidisciplinary team diagnosis is important for all individuals with ILD.

## Abbreviations

CT	Computerised tomography
CTD-ILD	Connective tissue disease related ILD
DLCO	Diffusing capacity of the lung for carbon monoxide
HP	Hypersensitivity pneumonitis
HRCT	High resolution computerised tomography
ILD	Interstitial lung disease
IPF	Idiopathic pulmonary fibrosis
MDT	Multidisciplinary team
NSIP	Non-specific interstitial pneumonitis
UIP	Usual Interstitial pneumonia
